# Economical synthesis of γ-cyclodextrin catalyzed by oriented cyclodextrin glycosyltransferase displayed on bacterial polyhydroxyalkanoate nanogranules

**DOI:** 10.1186/s12934-023-02191-2

**Published:** 2023-09-13

**Authors:** Menglu Duan, Yan Wang, Dan Tan, Guowu Yang, Yuan Deng, Ganqiao Ran, Jiao Li

**Affiliations:** 1 Shaanxi Institute of Microbiology, No. 76 Xi Ying Road, Xi’an, 710043 Shaanxi Province China; 2 Bio-Agriculture Institute of Shaanxi, Xi’an, 710069 China; 3 Shaanxi Key Laboratory of Qinling Ecological Security, Shaanxi Institute of Microbiology, Xi’an, 710043 China; 4 Key Laboratory of Biomedical Information Engineering of Ministry of Education, School of Life Science and Technology, Xi’an Jiaotong University, Xi’an, 710049 China

**Keywords:** γ-CGTase, γ-CD, Polyhydroxyalkanoates, Nanogranules, Immobilization

## Abstract

**Background:**

The advantages of γ-cyclodextrin (γ-CD) include its high solubility, ability to form inclusion complexes with various poorly water-soluble molecules, and favorable toxicological profile; thus, γ-CD is an attractive functional excipient widely used in many industrial settings. Unfortunately, the high cost of γ-CD caused by the low activity and stability of γ-cyclodextrin glycosyltransferase (γ-CGTase) has hampered large-scale production and application.

**Results:**

This study reports the in vivo one-step production of immobilized γ-CGTase decorated on the surface of polyhydroxyalkanoate (PHA) nanogranules by the N-terminal fusion of γ-CGTase to PHA synthase via a designed linker. The immobilized γ-CGTase-PHA nanogranules showed outstanding cyclization activity of 61.25 ± 3.94 U/mg (γ-CGTase protein) and hydrolysis activity of 36,273.99 ± 1892.49 U/mg, 44.74% and 18.83% higher than that of free γ-CGTase, respectively. The nanogranules also exhibited wider optimal pH (cyclization activity 7.0–9.0, hydrolysis activity 10.0–11.0) and temperature (55–60 °C) ranges and remarkable thermo- and pH-stability, expanding its utility to adapt to wider and more severe reaction conditions than the free enzyme. A high yield of CDs (22.73%) converted from starch and a high ratio (90.86%) of γ-CD in the catalysate were achieved at pH 9.0 and 50 °C for 10 h with 1 mmol/L K^+^, Ca^2+^, and Mg^2+^ added to the reaction system. Moreover, γ-CGTase-PHA beads can be used at least eight times, retaining 82.04% of its initial hydrolysis activity and 75.73% of its initial cyclization activity.

**Conclusions:**

This study provides a promising nanobiocatalyst for the cost-efficient production of γ-CD, which could greatly facilitate process control and economize the production cost.

**Supplementary Information:**

The online version contains supplementary material available at 10.1186/s12934-023-02191-2.

## Background

Cyclodextrins (CDs) are cyclic α-1,4-glucans produced from low-cost starch resources catalyzed by cyclodextrin glycosyltransferase (CGTase, EC 2.4.1.19) and used as solubilizing agent and stabilizer in various pharmaceutical and food products [1, 2]. The enzymatic products are usually mixtures of CDs containing 6 (α-CD), 7 (β-CD), or 8 (γ-CD) glucose units. CDs exhibit a hydrophilic outer surface and a non-polar/hydrophilic cavity that encapsulates the hydrophobic guest molecules, forming so-called inclusion complexes that can increase the efficacy of the active ingredient in biological and pharmaceutical formulations [3].

In comparison with α-CD and β-CD, γ-CD has the largest hydrophobic cavity, highest water solubility, and the most favorable toxicological profile. Therefore, there is a growing demand for γ-CD in many industrial settings [4]. However, the commercial production of γ-CD has been limited due to the lack of a favorable γ-CGTase with high specificity, high stability, and low cost [5]. Although several attempts have been made to screen high-specific γ-CGTase-producing bacterial strains, free γ-CGTases are sensitive to elevated temperatures and high pH reaction conditions, which can result in a decrease or even the loss of enzymatic activity [6, 7]. Furthermore, free enzymes cannot be recycled and reused after the catalytic reaction, further increasing production costs. Moreover, the purification of free γ-CGTase is time-consuming and costly, involving complex processes such as protein salting out, ultrafiltration, and size exclusion chromatography [8, 9]. Immobilizing γ-CGTase onto suitable supports to improve enzyme stability and reusability in γ-CD production can help solve the aforementioned issues.

To date, CGTase has been immobilized onto a wide range of natural and synthetic supports through different approaches, including surface adsorption, covalent binding, encapsulation, and cross-linking. CGTase from *Amphibacillus* sp. NPST-10 has been covalently immobilized onto amino-functionalized magnetic double mesoporous core–shell silica nanospheres (mag@d-SiO_2_@m-SiO_2_–NH_2_). Compared with the free enzyme, the immobilized CGTase showed significantly improved thermal and pH stability and retained 56% of its initial activity after ten successive cycles [10]. Zhang et al. described a recombinant CGTase immobilized on polydopamine–Fe_3_O_4_ (PDA–Fe_3_O_4_) nanoparticles that exhibited good thermal and operational stabilities and retained 19.1% β-activity after nine uses [11]. Other studies have immobilized CGTase from different species on other supports to improve reusability, including resin, glyoxyl–agarose, calcium alginate beads, cellulose nanofibers, and vegetable sponges [12–16]. Nevertheless, all of these conventional immobilization strategies are associated with numerous drawbacks, including a labor-intensive and complicated production process, prominently reduced activity of CGTase, and increased mass transfer limitations. Therefore, there is substantial interest in incorporating CGTase onto a suitable carrier to simplify the immobilization process and reduce production costs [17].

Polyhydroxyalkanoate (PHA) nanogranules, also referred to as bioplastics or biopolyesters, are naturally occurring inclusions comprising (R)-3-hydroxy fatty acids and used as intracellular carbon and energy storage by various bacteria under unbalanced nutrient conditions [18, 19]. These nanogranules comprise a spherical hydrophobic polyester core and a protein shell on their surface, such as PHA synthase (PhaC), phasins (PhaP), and depolymerases (PhaZ). The PHA biosynthetic pathway involves three key enzymes, β-ketothiolase (PhaA), acetoacetyl-CoA reductase (PhaB), and PhaC, together comprising the PhaABC operon. Heterogeneous organisms co-expressing these three enzymes can synthesize PHA granules [20]. Recently, the intracellular formation of PHA granules with covalently linked surface-associated proteins from *Ralstonia eutropha* has been exploited as a novel strategy for oriented enzyme immobilization. Fusing the enzyme of interest to PhaC could result in the one-step synthesis of PHA nanoparticles with the active enzyme displayed on their surface [21, 22]. This approach to one-step enzyme immobilization has produced higher enzyme activities and product levels than traditional immobilization techniques, and the products can be easily isolated from bacterial hosts by cell disruption and centrifugation [19, 23]. Hence, the one-step immobilization strategy represents an exciting new concept for enzyme immobilization that promises the cost-effective production of improved industrial biocatalysts.

**Fig. 1:**
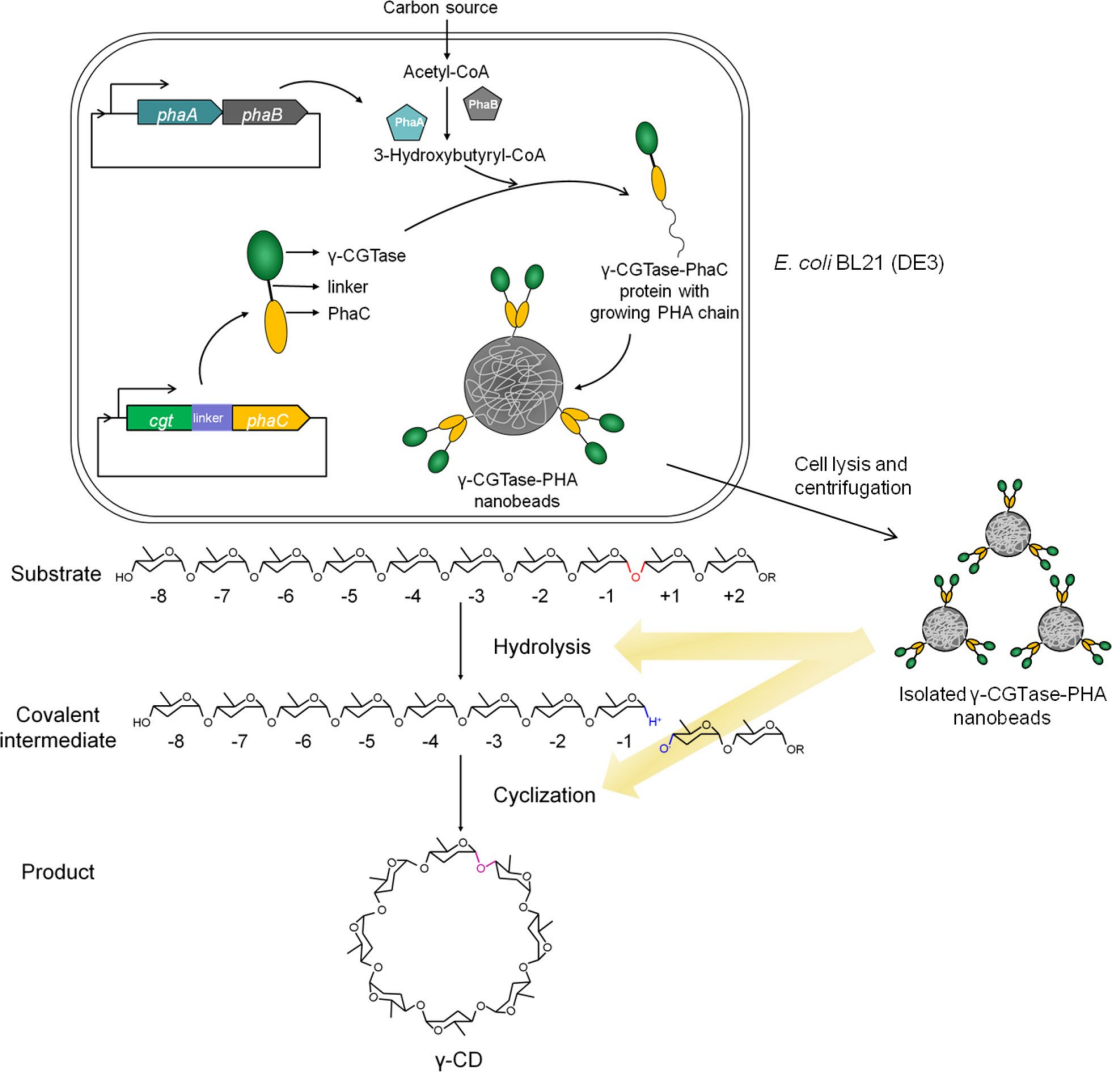
Synthesis scheme of γ-CD by one-step oriented immobilization of γ-CGTase on PHA nanogranules

In this study, a novel biocatalyst for the immobilization of γ-CGTase on the surface of PHA nanogranules was investigated by direct translational fusion of the γ-CGTase to PhaC. The co-expression plasmid pCDFD-ABC-cgt was designed and constructed for the first time and transformed into *Escherichia coli* (*E. coli*) BL21 (DE3), and the functional γ-CGTase-PHA nano-granules were synthesized in vivo. Furthermore, the fused protein γ-CGTase-PhaC on the surface of PHA nanogranules was identified and quantified by mass spectrometry (MS)-based quantitative proteomics. The enzymatic, catalytic, and reutilization properties of the nanogranules were then characterized. Finally, the nanogranules were employed for the enzymatic synthesis of γ-CD (Fig. 1).

## Results

### Expression and purification of free γ-CGTase in recombinant *E. coli* BL21 (DE3)

**Fig. 2:**
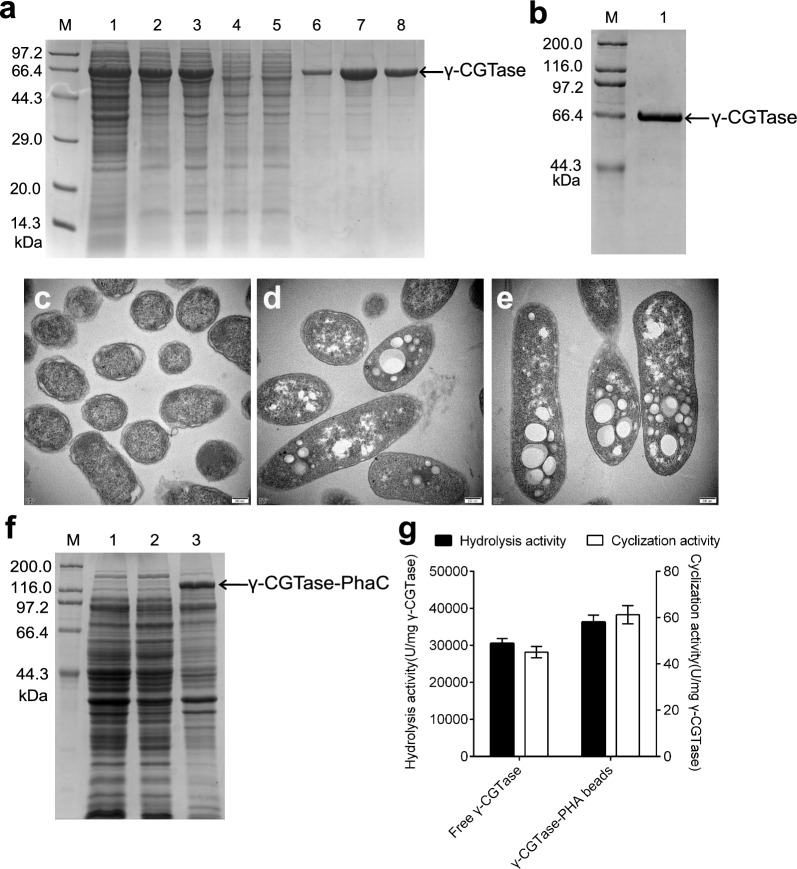
Expression of free γ-CGTase and identification of PHA-based nanobeads generated by co-expression of γ-CGTase and PhaC. **a** SDS–PAGE analysis of free γ-CGTase purified by the α-CD-Sepharose 6B affinity column. Lane M, protein ladder; Lane 1, the lytic supernatant of the recombinant *E. coli* BL21 (DE3) strain harboring the pET22b (+)-cgt plasmid after induction; Lane 2, resuspension of the precipitate obtained from the lytic supernatant (Lane 1) after adding 40% saturated ammonium sulfate solution; Lane 3, the protein solution of the resuspended precipitate (Lane 2) after desalting; Lanes 4 and 5, the flow-through of the desalting protein loaded onto the α-CD-Sepharose 6B affinity column; Lanes 6–8, the eluate obtained from the affinity column. **b** SDS–PAGE analysis of free γ-CGTase purified by size exclusion chromatography (SEC) equipped with a HiLoad 16/600 Superdex 200 pg column. Lane M, protein ladder; Lane 1, the purified free γ-CGTase eluted from the column**. c**–**e** Transmission electron microscopy analysis of unmodified *E. coli* cells (**c**), recombinant *E. coli* carrying the blank plasmid pCDFD-ABC (**d**), and recombinant *E. coli* carrying pCDFD-ABC-cgt (**e**) after induction. **f** SDS–PAGE analysis of γ-CGTase-PHA beads expressed by the recombinant *E. coli* BL21 (DE3) strain harboring the pCDFD-ABC-cgt vector. Lane M, protein ladder; Lane 1, non-induced strain; Lane 2, lytic supernatant of the IPTG-induced strain; Lane 3, lytic precipitation of the IPTG-induced strain. **g** Determination of the hydrolysis and cyclization activity of free γ-CGTase and immobilized γ-CGTase. Values are averages of results from triplicate trials; the error bars indicate the SD values

The *E. coli* BL21 (DE3) strain harboring the recombinant plasmid pET22b (+)-cgt was synthesized according to the biased codons of *E. coli* BL21 (DE3) and induced to produce free γ-CGTase by the addition of Isopropyl-β-d-1-thiogalactopyranoside (IPTG) as described in our previous study [24]. After the supernatant of cell lysis was precipitated by ammonium sulfate, the free γ-CGTase was purified by an α-CD-Sepharose 6B affinity column (Fig. 2a) and further purified using size exclusion chromatography (SEC) equipped with a Superdex 200 pg column (Fig. 2b). The apparent electrophoresis-pure γ-CGTase was observed as a single band with the expected molecular weight of 66 kDa (Fig. 2b), indicating that the free γ-CGTase produced had high purity.

### Display of γ-CGTase on the surface of PHA nanogranules via N-terminal fusion to PhaC

To immobilize γ-CGTase on the surface PHA nanogranules, the PHA biosynthetic pathway from *Ralstonia eutropha* H16 was first introduced to the *E. coli* BL21 (DE3) strain using the pCDFD-ABC-cgt plasmid containing two T7 promoters. The *phaAB* genes encoding PhaA and PhaB, which are essential for the precursor supply to form PHA nanogranules, were inserted into the pCDFDuet-1 plasmid under the control of the first T7 promoter. Then, the *cgt-linker* sequence containing a 5′ *cgt* gene encoding the γ-CGTase from *Bacillus clarkii* 7364 without its stop codon and including a 3′ *linker* gene encoding the peptide (GGGSGGGSGGGS) was inserted downstream of the second T7 promoter, generating the pCDFD-AB-cgt plasmid. Finally, the *phaC* gene without its start codon was inserted into the pCDFD-AB-cgt to generate the co-expression plasmid pCDFD-ABC-cgt. The DNA Sanger sequencing and digestion results confirmed the correct plasmid sequence. Then the recombinant *E. coli* BL21 (DE3) strain harboring the pCDFD-ABC-cgt vector was constructed by transforming the verified recombinant plasmid into *E. coli* BL21 (DE3) cells, which were then cultured in a modified TB-MM medium to synthesize PHA granules.

The formation of PHA granules in recombinant *E. coli* was confirmed directly by TEM. As shown in Fig. 2c–e, the recombinant *E. coli* BL21 (DE3) cells harboring the pCDFD-ABC-cgt or blank pCDFD-ABC plasmids contained white PHA granules at diameters ranging from 30 to 280 nm, whereas no granules were observed in the wild-type cells. The protein expression profiles of the recombinant *E. coli* harboring the plasmid pCDFD-ABC-cgt before and after induction were determined by SDS–PAGE (Fig. 2f), and a new band corresponding to the γ-CGTase-PhaC fusion protein with the expected apparent molecular weight of 142 kDa was observed in the induced cell precipitation, suggesting that γ-CGTase was successfully displayed on the surface of PHA granules by N-terminal fusion with PhaC. The identity and quantity of this fusion protein associated with the PHA granules were further confirmed by LC–MS/MS.

### Identification and absolute quantification of fused γ-CGTase on PHA nanogranules and determination of its activity

The incorporation of differential stable isotopes into samples is an accurate and reliable method for protein idenfication and absolute quantification [25]. Here, LC–MS/MS was conducted on the mixture of isotopic labeled total PHA granule-associated protein digest and purified free γ-CGTase digest. To obtain a complete proteome of the surface proteins attached to PHA nanogranules, the customized database including PhaA, PhaB, and γ-CGTase-PhaC fusion protein or free γ-CGTase was used to search the tandem mass spectra for accurate protein identification. Furthermore, the relative ion abundances of the protein digests were compared to quantify the anchored proteins. As shown in Additional file 1: Table S1, over 200 proteins were confidently identified (protein score > 30, FDR < 1%) on the surface of PHA granules. The most abundant proteins attached on PHA granules were γ-CGTase-PhaC, PhaA, and PhaB, of which the γ-CGTase-PhaC fusion protein comprised approximately 0.568% of the total weight of purified nanobeads [0.568% = (1/0.175972) (light/heavy) × (1:1000) (the ratio of dilution times, light/heavy)]. The ratio of fused protein was higher than the 0.472% described previously. Meanwhile, other abundant proteins on the PHA granule surface were identified as the major contaminating proteins, including the elongation factor Tu, porin gram-negative type, outer membrane protein A, and ribosomal protein L14.

CGTase is a member of the α-amylase family of glycosyl hydrolases (family 13), an important group of starch-converting enzymes. Unlike normal amylases, which generally hydrolyze glycosidic bonds in starch and glycogen, CGTase activity comprises four reactions (cyclization, coupling, disproportionation, and hydrolysis) [2]. The enzyme primarily catalyzes cyclization reactions, and the hydrolysis activity of CGTase also plays an essential role in the manufacturing of CDs because hydrolyzing starch by other α-amylases would generate maltoses or other oligosaccharides that are challenging to use to synthesize CDs [26]. However, the small molecules produced by CGTase treatment are mainly CDs [27]. Therefore, in this study, the γ-CGTase self-assembled on the surface of PHA granules was revealed by detecting its ability to hydrolyze starch (hydrolysis activity) and synthesize γ-CD (cyclization activity).

According to the LC–MS/MS quantification result presented above, the fused γ-CGTase contributed to 0.568% (w/w) of the dry beads; thus, the amount of fusion protein in 10 mg dry beads was equivalent to 0.0568 mg free γ-CGTase powder. To determine the specific activities of fused γ-CGTase, 10 mg dry beads and 0.0568 mg free γ-CGTase were independently added to a gelatinized starch solution to produce a catalytic reaction. As shown in Fig. 2g, the cyclization activity of the immobilized γ-CGTase on the surface of the PHA beads was 61.25 ± 3.94 U/mg γ-CGTase protein, 44.74% higher than that of free γ-CGTase (45.08 ± 2.44 U/mg protein). In addition, the hydrolysis activity of γ-CGTase-PHA granules (36,273.99 ± 1892.49 U/mg protein) was 18.83% higher than that of free γ-CGTase (30,527.03 ± 1321.01 U/mg protein), suggesting that display on PHA is a promising strategy to extend the utility of the enzyme.

### Optimization of culture conditions for recombinant *E. coli* BL21 (DE3) generating γ-CGTase-PHA nanogranules

**Fig. 3:**
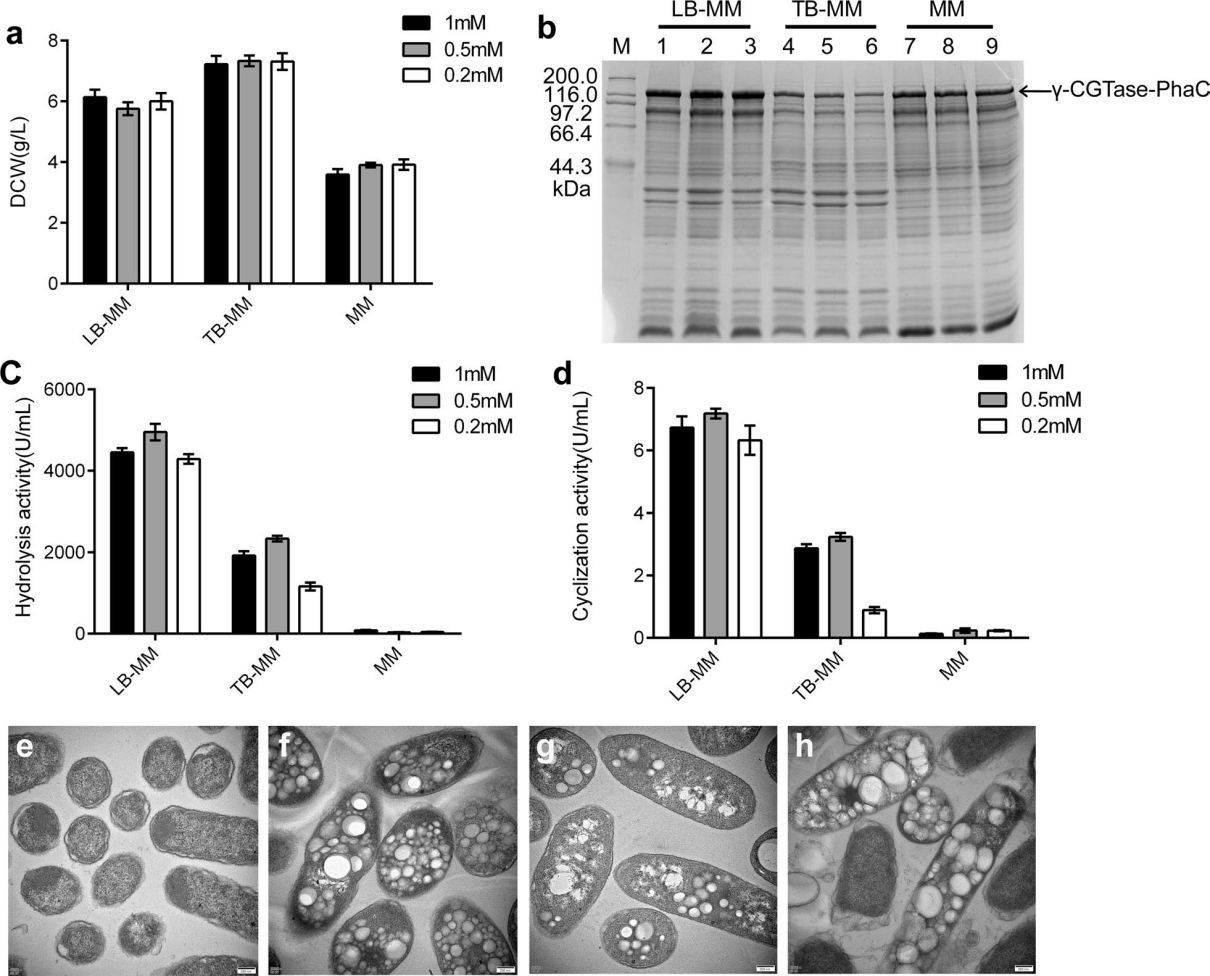
The effects of different culture media and different concentrations of IPTG on the production of γ-CGTase-PHA nanogranules by recombinant *E. coli* BL21 (DE3). **a** Comparison of cell growth in different shake-flask cultures supplemented with different concentrations of IPTG. **b** SDS–PAGE analysis of γ-CGTase-PHA beads expressed by recombinant *E. coli* BL21 (DE3) cultured in different shake-flask cultures supplemented with different concentrations. Lane M, protein ladder; Lanes 1–3, LB medium supplemented with 1 mM, 0.5 mM, 0.2 mM IPTG, respectively**;** Lanes 2–4, TB medium supplemented with 1 mM, 0.5 mM, or 0.2 mM IPTG, respectively**;** Lanes 5–7, MM medium supplemented with 1 mM, 0.5 mM, or 0.2 mM IPTG, respectively**. c**, **d** Comparison of hydrolysis activities (**c**) and cyclization activities (**d**) of γ-CGTase-PHA beads in different shake-flask cultures supplemented with different concentrations of IPTG. **e–h** Transmission electron microscopy analysis of unmodified *E. coli* cells (**e**), recombinant *E. coli* carrying pCDFD-ABC-cgt cultured in LB (**f**), TB (**g**), and MM (**h**) medium after 0.5 mM IPTG induction

The culture medium of the recombinant *E. coli* BL21 (DE3) cells harboring pCDFD-ABC-cgt and the concentration of IPTG in the medium were optimized to increase the yield of active γ-CGTase-PHA nanogranules. Component II of MM medium was replaced with the components of LB and TB medium (yielding LB-MM and TB-MM, respectively). Then the expression of the γ-CGTase-PhaC protein and the activity levels of the γ-CGTase-PHA nanogranules in recombinant cells cultured in the three different media were determined. As shown in Fig. 3a, the highest dry cell weight (DCW) of bacteria was harvested from TB-MM medium reaching 7.33 g/L; the yield was ~ 20% lower with LB-MM medium. However, the synthesis of PHA granules (Fig. 3g) and the surface protein γ-CGTase-PhaC (Fig. 3b) was lowest in recombinant bacteria obtained from TB-MM medium, potentially because PHA granules usually tend to be synthesized in bacteria under carbon excess and nitrogen-limiting conditions, rather than in medium with extremely abundant nitrogen [22]. Notably, the quantity of PHA granules and the surface protein was higher in MM medium than in TB medium (Fig. 3b and h); however, these nanogranules showed almost no hydrolysis or cyclization activities (Fig. 3c and d). As shown in Fig. 3b–d, the recombinant *E. coli* BL21 (DE3) cells cultured in LB-MM medium expressed the highest level of γ-CGTase-PhaC fused protein and displayed the highest γ-CGTase hydrolysis and cyclization activities. The TEM images confirmed that the cells cultured in LB-MM medium produced the greatest number of PHA granules (Fig. 3f). These results suggest that LB-MM medium is the optimal medium for promoting the production of active γ-CGTase-PHA nanogranules by recombinant *E. coli* BL21 (DE3).

Meanwhile, IPTG was added to the medium at the final concentration of 0.2 mM, 0.5 mM, or 1 mM. As shown in Fig. 3a, the final concentration of IPTG had no significant effect on bacterial DCW. However, 0.5 mM IPTG greatly improved the hydrolysis (4951.79 U/mL) and cyclization (7.18 U/mL) activities of the immobilized γ-CGTase (Fig. 3c and d). In conclusion, LB-MM medium supplemented with 0.5 mM IPTG is the most favorable condition for synthesizing active γ-CGTase-PHA nanogranules in recombinant *E. coli* BL21 (DE3).

### Enzymatic characteristics of γ-CGTase immobilized on PHA beads

**Fig. 4:**
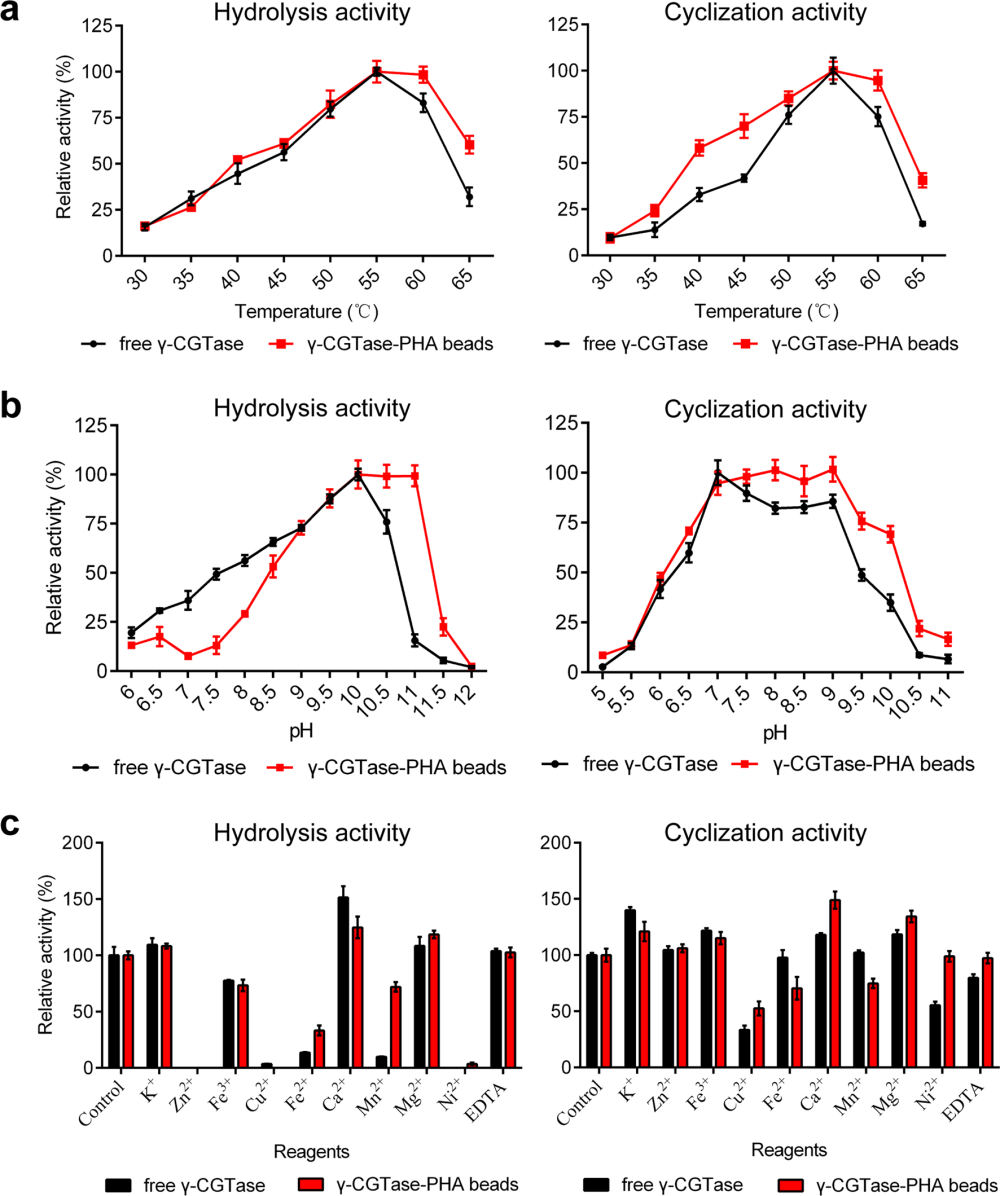
Enzymatic characteristics of free γ-CGTase and γ-CGTase-PHA beads. The effect of temperature (**a**), pH (**b**), and various metal ions (**c**) on the hydrolysis activity and cyclization activity of free γ-CGTase and immobilized γ-CGTase. The data were obtained from triplicate independent trials; the error bars indicate the SD values

The temperature and pH dependency profiles of the immobilized γ-CGTase and the free enzyme were subsequently investigated. The effects of temperature on hydrolysis and cyclization activities were monitored from 30 °C to 65 °C. As shown in Fig. 4a, the free enzyme exhibited maximal hydrolysis and cyclization activity at 55 °C and rapidly inactivated when the reaction temperatures exceeded 60 °C; however, the hydrolysis and cyclization activities of immobilized γ-CGTase were optimally active at a wider range (55–60 °C) than that observed with the free enzyme.

As revealed by the observations of characteristics at different pH values, the immobilization of γ-CGTase on PHA granules can broaden the range of pH adaptation. The maximal hydrolysis activity of immobilized and free γ-CGTase was reached at pH 10.0–11.0 and pH 10.0, respectively (Fig. 4b); the cyclization activity of the immobilized and free γ-CGTase was optimally active at pH 7.0–9.0 and pH 7.0, respectively (Fig. 4b). These results indicate that the immobilized γ-CGTase had a wider optimal pH range than that of free γ-CGTase. It is important to note that immobilized γ-CGTase maintained almost 100% cyclization activity at pH 9.0 and 70% cyclization activity at pH 10.0, while free γ-CGTase only exhibited 86% activity and 35% activity, respectively, at these pH levels. Considering that the hydrolysis and cyclization reactions occur simultaneously during the γ-CD production process, and the specificity of CGTase is much higher in alkaline reaction conditions than at neutral and acidic pH values, γ-CGTase-PHA nanogranules are better suited than the free enzyme for the complicated production processes of γ-CD.

The γ-CGTase-PHA granules were then subjected to Tris–HCl buffer (50 mM, pH 6.0) supplemented with various metal ions and reagents. As shown in Fig. 4c, the addition of EDTA to the reaction system slightly inhibited the hydrolysis activity of both immobilized and free γ-CGTase, Ca^2+^ could dramatically increase the enzyme activity, and K^+^ and Mg^2+^ had relatively weak promoting effects. This result may be because the activation of most members of the alpha-amylase family were activated by Ca^2+^ and inhibited by EDTA [28]. By contrast, other metal ions, such as Zn^2+^, Fe^3+^, Cu^2+^, Fe^2+^, Mn^2+^, and Ni^2+^, significantly inhibited the hydrolysis activity of both immobilized and free γ-CGTase, especially Zn^2+^, which completely inhibited the enzyme activity. Moreover, K^+^, Fe^3+^, Ca^2+^, and Mg^2+^ obviously enhanced the cyclization activity of both immobilized and free γ-CGTase, while other metal ions and reagents had no significant effect or an inhibitory effect on enzyme activity (Fig. 4c). Considering the effects of all the above metal ions on the hydrolysis and cyclization activities, 1 mmol/L K^+^, Ca^2+^, and Mg^2+^ can be added to promote the forward conversion reaction of starch to γ-CD.

### Thermostability of γ-CGTase immobilized on PHA nanogranules

**Fig. 5:**
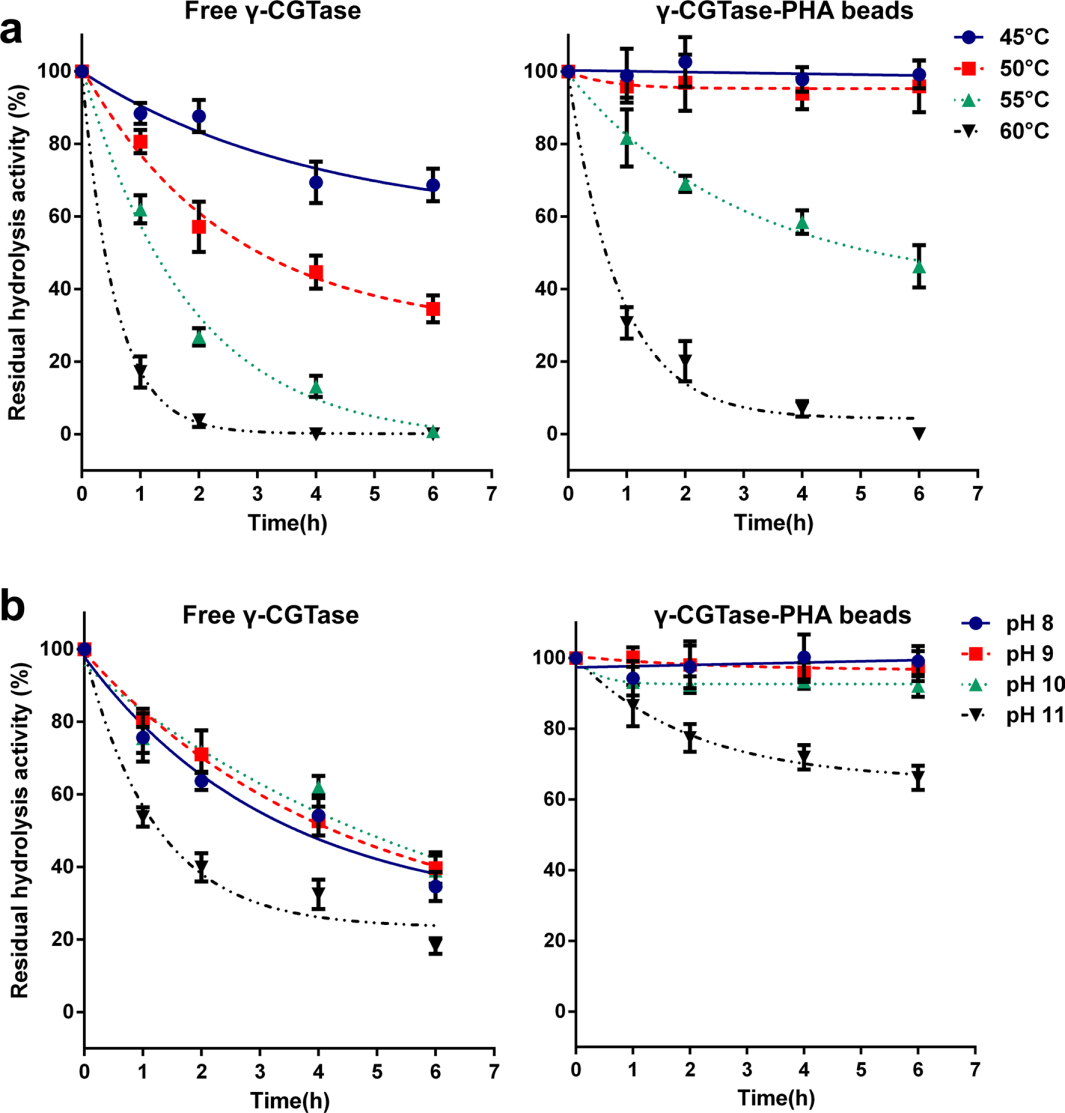
Thermal (**a**) and pH stability (**b**) of the hydrolysis activity of free γ-CGTase and γ-CGTase-PHA beads. The data were obtained from triplicate independent trials; the error bars indicate the SD values

The thermal stabilities of the two enzymes were tested by monitoring the residual activity of the immobilized and free γ-CGTase after incubation at 45 °C, 50 °C, 55 °C, and 60 °C for 6 h in Tris–HCl buffer (50 mM, pH 9.0) with 1 mmol/L K^+^, Ca^2+^, and Mg^2+^ added at the indicated time intervals. As shown in Fig. 5a and b, the immobilized γ-CGTase exhibited excellent thermostability regarding hydrolysis and cyclization activities and retained almost full initial activity after a 6 h incubation at 45 °C and 50 °C. However, the free enzyme only maintained 69% of the initial hydrolysis activity at 45 °C and was almost completely deactivated at 50 °C. Similarly, the cyclization activity of free γ-CGTase only maintained 65% and 11% of the initial activity at 45 °C and 50 °C, respectively (Fig. 5b). These results were in agreement with the broader temperature tolerance shown in Fig. 4a. Notably, the stability of γ-CGTase-PHA nanogranules decreased significantly at 55 °C, with a 54% reduction in hydrolysis activity and a 41% reduction in cyclization activity. Therefore, the biocatalysis of γ-CGTase-PHA nanogranules for γ-CD production should not be performed at temperatures exceeding 50 °C.

### pH stability of γ-CGTase immobilized on PHA nanogranules

**Fig. 6:**
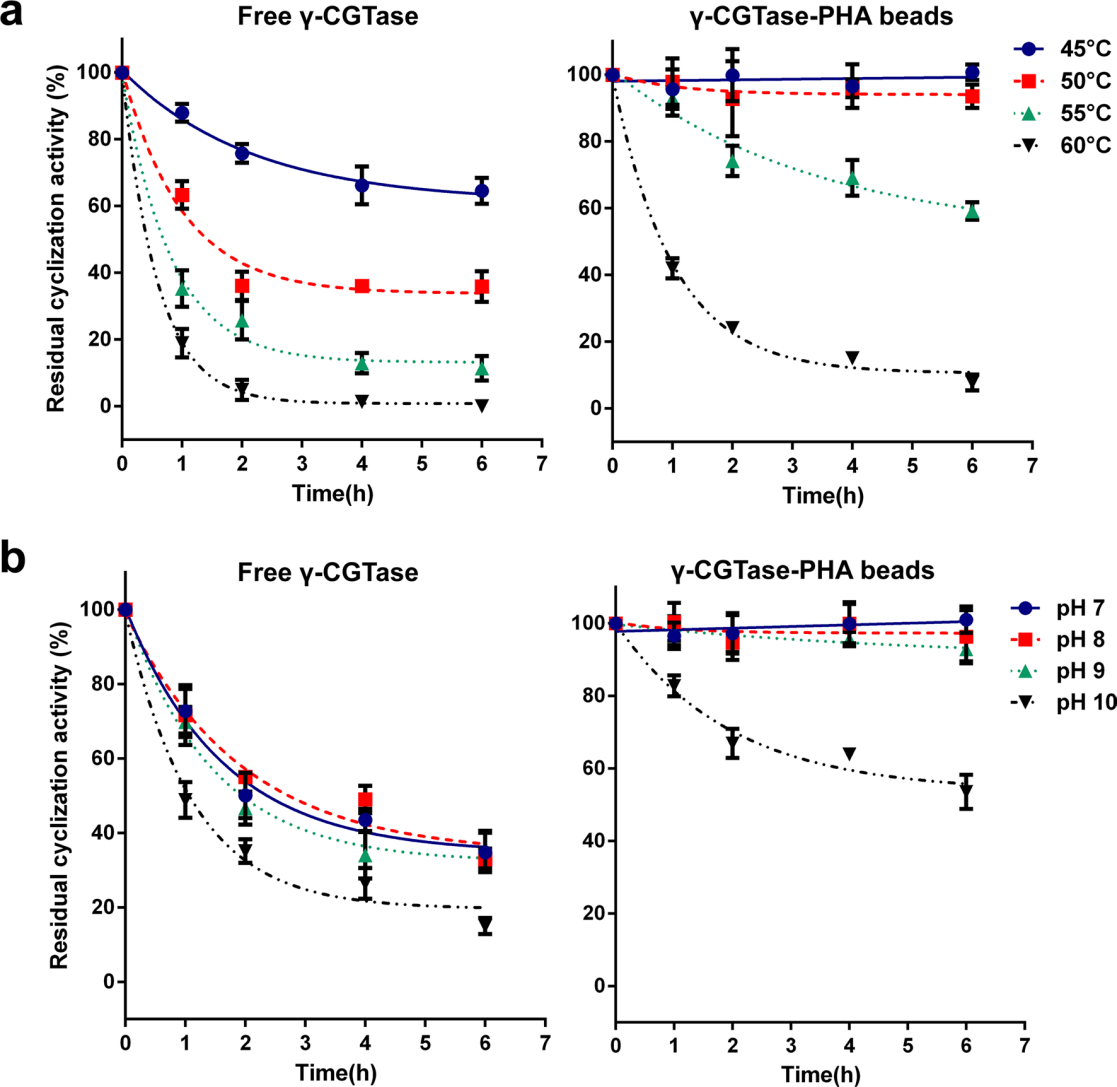
Thermal (**a**) and pH stability (**b**) of the cyclization activity of free γ-CGTase and γ-CGTase-PHA beads. The data were obtained from triplicate independent trials; the error bars indicate the SD values

The pH stability of γ-CGTase activity was significantly enhanced in neutral and alkaline conditions when γ-CGTase was attached to the surface of PHA granules. The free γ-CGTase lost ∼60% of its initial hydrolysis activity after 6 h of incubation at 55 °C in buffers supplemented with 1 mmol/L K^+^, Ca^2+^, and Mg^2+^ at pH 8.0–10.0, while the immobilized enzyme was extremely stable at pH 8.0–10.0 with no significant loss in comparison with the initial activity levels (Fig. 6a). Moreover, the cyclization activity of immobilized γ-CGTase exhibited similar excellent stability in neutral and alkaline conditions (pH 7.0–9.0); however, the free enzyme lost ∼70% of its initial activity under the same conditions (Fig. 6b). These stable properties of immobilized γ-CGTase make it superior to the free enzyme and other γ-CGTases and widen its applicability during the γ-CD production process.

### Determination of the synthesis conditions of γ-CGTase-PHA nanogranules in γ-CD production catalysis

**Fig. 7:**
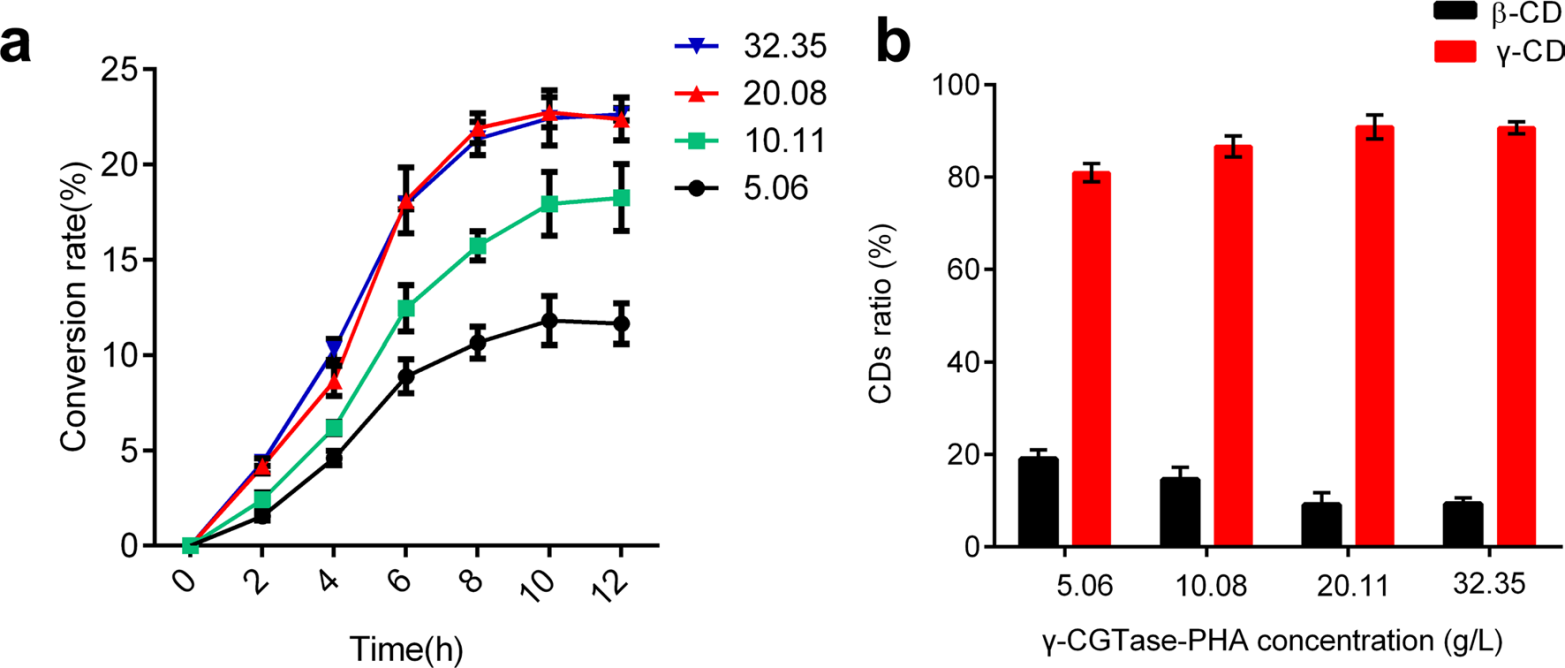
The effect of the γ-CGTase-PHA concentration on the yield of CDs (**a**) and the ratio of γ-CD in the products (**b**). The data were obtained from triplicate independent trials; the error bars indicate the SD values

According to the enzymatic characteristics of γ-CGTase-PHA beads, the levels of various factors, including the catalysis temperature, pH, and metal irons affecting enzymatic reactions, have been established. In this study, γ-CGTase-PHA beads were added to a 2% (w/v) gelatinized starch solution at different final concentrations and catalyzed the reaction for 12 h. The yield and ratio of γ-CD in the products were measured hourly. As shown in Fig. 7a, the yield of soluble starch converted into γ-CD increased rapidly, especially in the first 10 h, and subsequently remained at equilibrium at 10–12 h. The maximal yield of γ-CD from starch was 11.65%, 18.26%, 22.05%, and 22.63% with 5.06, 10.11, 20.08, and 32.35 g/L of γ-CGTase-PHA beads treated, respectively, indicating that 20.08 g/L γ-CGTase-PHA beads were already a saturating biocatalyst load for the conversion of 2% (w/v) starch to γ-CD. In addition, the ratio of γ-CD in the products achieved 90.67% when 20.08 g/L nanogranules were added to the reaction system. When the concentration of γ-CGTase-PHA beads was further increased (> 20.08 g/L), the ratio of γ-CD was not further improved (Fig. 7b). This saturation concentration of γ-CGTase-PHA beads was also in agreement with that shown in Fig. 7a. Our results suggest that increasing the quantity of γ-CGTase-PHA beads in the reaction system could improve the yield and the ratio of γ-CD in the products. Consequently, the optimal conditions for producing γ-CD using γ-CGTase-PHA nanogranules were determined to be 1 mmol/L K^+^, 1 mmol/L Ca^2+^, 1 mmol/L Mg^2+^, 20.08 g/L γ-CGTase-PHA nanogranules, pH 9.0, and 50 °C, with rotating for 10 h.

### γ-CD production in repetitive batches by recycling γ-CGTase-PHA nanogranules

**Fig. 8:**
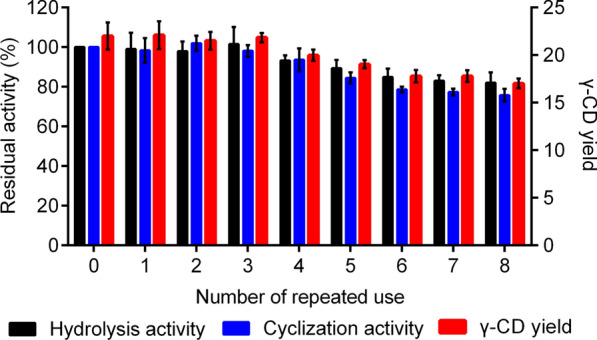
Reusability of the γ-CGTase-PHA beads. The residual activity was defined as the ratio of activity after each cycle to the initial activity. The data were obtained from triplicate independent trials; the error bars indicate the SD values

γ-CD production by γ-CGTase displayed on PHA nanogranules was performed in eight repetitive batches under the optimized conditions. After each batch, the nanogranules were recovered by centrifugation (8000 ×*g*, 20 min, 4 °C) and resuspended in a fresh reaction mixture for another cycle of biocatalysis. As shown in Fig. 8, the yield was 22.00% after the first batch, remained considerably stable during the first three batches, and showed a gentle decline of 22.64% after the eighth batch. In addition, 82.04% of the initial hydrolysis activity and 75.73% of the initial cyclization activity remained after eight repeated uses of the immobilized γ-CGTase, corresponding to a relatively stable γ-CD yield per cycle, demonstrating excellent reusability. These results indicate that γ-CGTase-PHA nanogranules are promising biocatalysts for the cost-effective production of γ-CD.

## Discussion

γ-CD has great potential for application in foods, cosmetics, medicine, textiles, and environmental modifications due to its favorable properties on stabilizing and solubilizing guest molecules [1, 29]. However, the poor specificity and low activity of γ-CGTase lead to the inefficient and high-cost production of γ-CD. For instance, 79% of the total CDs were γ-CD in pre-gelatinized potato starch catalyzed by γ-CGTase from an alkalophilic *Bacillus clarkii* 7364 [6]. Hirano et al. reported the production of a novel γ-CGTase from alkalophilic *Bacillus sp.* G-825–6, which produced γ- and β-CD at a ratio exceeding 4.7 [30]. However, the aforementioned CGTases showed extremely low activities, rendering them unsuitable for the industrial production of γ-CD. The *E. coli* BL21 (DE3) expression strain harboring the recombinant plasmid pET22b (+)-cgt was designed and synthesized in our previous study to enhance the expression and activity of γ-CGTase from *Bacillus clarkii* 7364, and its culture conditions were optimized to enhance γ-CGTase activity 2.83-fold. Further analyses of the products converted from starch revealed that the majority (90.44%) of product CDs were the γ form, nearly 11% higher than the wild enzyme. However, the free γ-CGTase showed poor thermal stability, exceeding a 50% loss of hydrolysis and cyclization activities after only 20 min treatment at the optimal temperature (55 °C) [24]. Thus, our purpose in this study was to develop a robust and reused biocatalytic system for cost-effective production of γ-CD.

A simple one-step method to obtain enzymes displayed on PHA beads was exploited as a cost-effective strategy for enzyme immobilization, superior to the traditional adsorption, embedding, or chemical cross-linking strategies. The nano-biocatalyst showed excellent properties, including improved activity, remarkable thermo- and pH-stability, and excellent reusability [31]. This study first demonstrated that the functional display of the γ-CGTase from *Bacillus clarkii* 7364 on the surface of PHA granules could be achieved by the N-terminal fusion of γ-CGTase to PhaC via a designed linker (G3SG3SG3SG3S). The recombinant *E. coli* BL21 (DE3) strain harboring the plasmid pCDFD-ABC-cgt was designed and constructed, and SDS–PAGE and TEM analyses of the recombinant cells confirmed the abundant immobilization of γ-CGTase on the surface of the PHA beads. LC–MS/MS analysis indicated that the fused protein γ-CGTase-PhaC occupied 0.568% (w/w) of the total weight of the purified granules. The culture conditions of recombinant bacteria were also optimized to improve the expression of PHA granules and the fused protein γ-CGTase-PhaC on their surface. In this study, the recombinant cells cultured in MM medium produced numerous PHA granules and the fused γ-CGTase-PhaC; however, these nanogranules showed almost no hydrolysis or cyclization activities. The modified TB-MM medium significantly enhanced the enzyme activity but greatly reduced the number of granules synthesized by recombinant cells. By contrast, recombinant cells cultured in modified LB-MM medium produced the highest γ-CGTase-PHA nanogranule yield, and these nanogranules demonstrated the highest activity compared with those obtained from cells cultured in the other media. This effect may be because PHA granules tend to be synthesized in bacteria cultured under carbon excess and nitrogen-limiting conditions, such as basal MM medium [32], but the expression of active γ-CGTase heavily depends on a high concentration of nitrogen in the medium. Our previous study revealed that nitrogen deficiency could result in the correct protein conformation and lead to a sharp decrease in enzyme activity [24]. Thus, the carbon and nitrogen source ratio in the culture medium is essential for balancing the processes of PHA granule formation and activated γ-CGTase expression.

The enzymatic activity, stability, and reusability of γ-CGTase-PHA nanogranules were further characterized to investigate the advantages of the nanogranules as a novel nanobiocatalyst. The cyclization and hydrolysis activities of immobilized γ-CGTase were 44.7% and 18.83% higher than those of the free enzyme, respectively. Similar results were reported by Tan et al. and Li et al. Tan et al. discovered that the specific MP activity of the tyrosinase *Verrucomicrobium spinosum* (TyrVs) immobilized on PHA was 3.19-fold higher than that of free TyrVs [33]. Li et al. found an increased affinity between the OPAA4301-PHA nanogranules and the substrate, with the K_cat_/K_m_ value of PHA nanogranules twice that of the free enzyme [34]. This finding can be attributed to the favorable interaction between the carrier and the enzyme, resulting in enzyme folding into the optimum conformation on the surface of the PHA granules, thus increasing the enzyme activity [21, 23]. Furthermore, the γ-CGTase-PHA nanobiocatalyst was found to possess a considerable specific activity of 347.9 U/g beads (dry weight), considerably higher than that of other immobilized CGTases reported in most recent studies, such as glyoxyl–agarose immobilized CGTase (27.38 U/g support) [13], cellulose nanofiber immobilized CGTase (159.34 U/g support) [14], and glutaraldehyde pre-activated silica immobilized CGTase (101.73 U/g support) [35]. This finding is likely because PhaC dictates a homogenous orientation of its fusion partner on the nanobeads’ surface, hence providing a maximum interaction with substrates and increased accessibility to substrates, leading to high specific activity of the immobilized enzymes if properly engineered [22, 36].

In addition, the immobilized γ-CGTase exhibited significantly improved thermostability and pH tolerance and wider optimal pH and temperature ranges than the free enzyme. The immobilized γ-CGTase showed a broader temperature tolerance (Fig. 4a) and robust activity at temperatures up to 50 °C, while the free enzyme was almost completely deactivated at 50 °C (Fig. 5a and b). The immobilized γ-CGTase also reached optimal hydrolysis activity at pH 10.0–11.0 and optimal cyclization activity at pH 7.0–10.0, representing a wider optimum range than that of the free enzyme (hydrolysis activity at pH 10.0, cyclization activity at pH 7.0). To the best of our knowledge, CGTases always show much higher specificity in alkaline reaction conditions than at neutral and acidic pH values [3, 6, 30, 37]; therefore, γ-CGTase-PHA nanogranules are better biocatalysts for enhancing the productivity of γ-CD. Importantly, PHA nanogranules decorated with γ-CGTase showed stably high hydrolysis at pH 8.0–10.0 and cyclization activity at pH 7.0–9.0, while the activity of the free enzyme decreased dramatically under the same conditions. Similar results were observed in studies on D-tagatose 3-epimerase (DTE) and organophosphorus hydrolyzing enzyme (OPAA4301) on PHA beads. Free DTE lost ∼60% of its initial activity after 24 h of incubation at pH 7.0–8.0, while there was no loss of immobilized DTE activity under the same conditions [38]. The enzyme OPAA4301 displayed on PHA granules also exhibited higher activities than the free enzyme under both acidic and alkaline conditions and better thermal stability and pH tolerance than the free-state enzyme [34]. The conformational perturbance of the enzyme displayed on PHA through covalent binding could make the enzyme structurally rigid, enhancing enzyme activity and stability [20, 22]. These properties of immobilized γ-CGTase could broaden its applicability, making it more robust and better suited for the complicated γ-CD production process.

A gelatinized starch solution was catalyzed using different concentrations of γ-CGTase-PHA beads under the optimized conditions to test γ-CD production. When the nanobead concentration reached 20.08 g/L, a considerable yield of 22.73% with a 90.86% ratio of γ-CD was achieved after a 10 h reaction. The ratio of γ-CD (90.86%, Fig. 7b) in the CDs produced by γ-CGTase-PHA beads was the highest among previously published immobilized CGTases, indicating that the difficulty and cost of subsequent separation and extraction of γ-CD could be greatly reduced. However, the yield (22.73%) of γ-CD was not the highest among the published immobilized CGTases; the γ-CD yield was lower than that obtained from immobilized β-CGTase on glutaraldehyde pre-activated silica (26% β-CD, pH 4.0, 70 °C) [35] and glyoxyl–agarose (29% β-CD, 85 °C) [13], but compared favorably with that of CGTases immobilized on other substances, such as aminated polyvinylchloride (PVC) (15.6%) [39] and resin (FE 4611) (14%) [12]. When the immobilized γ-CGTase level was further increased, the yield and the ratio of γ-CD at equilibrium were not further improved (Fig. 7a), potentially because the cyclizing activity of CGTase can be inhibited in the presence of high concentrations of product, which causes decreased enzyme productivity as the concentration of CDs in the reaction medium increases. Thus, in the batch production of CDs, the final equilibrium would not be shifted toward CD accumulation by higher concentrations of CGTase when the product CDs have already accumulated to a certain extent [15]. In future studies, the yield of starch to γ-CD will be elevated through the removal of product γ-CD with the addition of organic solvents, such as glycyrrhizic acid and cyclododecanone, to the reaction mixture and through continuous production processes, such as ultrafiltration systems and packed-bed reactors, in which the continuous inflow of starch and outflow of γ-CD may reduce the inhibition of γ-CGTase activity and shift the reaction balance toward the direction of γ-CD formation.

The γ-CGTase-PHA nanogranules also exhibited excellent reusability with only a slight decrease in both hydrolysis and cyclization activity after eight times of repeated use, sustaining a relatively stable yield of γ-CD (over 16%). As previously reported, the centrifugal recovery of nanogranules after each cycle resulted in a 3% reduction in comparison with the initial weight [38], representing one of the main causes of the decline in residual activity. The strong covalent interaction between the fused protein (γ-CGTase-PhaC) and the matrix (PHA nanogranules) prevents the enzyme from detachment and denaturation, demonstrating that γ-CGTase-PHA nanogranules are a very promising biocatalyst for the cost-effective production of γ-CD.

## Conclusion

In this study, a competitive approach for the one-step in vivo production of immobilized γ-CGTase displayed on bacterial PHA nanogranules was provided by the N-terminal fusion of γ-CGTase to PhaC via a designed linker. Our results indicate that immobilization on PHA significantly enhances the catalytic activity, thermo-stability, and pH-stability of γ-CGTase and broadens its optimal pH and temperature ranges, expanding its utility to adapt to harsher working conditions vs. that of the free enzyme. The immobilized γ-CGTase exhibited remarkable enzyme activity of 347.9 U/g beads, a high yield of CDs (22.73%) converted from starch, and a high resulting γ-CD ratio (90.86%). Moreover, γ-CGTase-PHA beads can be used eight times, retaining 82.04% of the initial hydrolysis activity and 75.73% of the initial cyclization activity, greatly facilitating the process control, productivity, and purification steps. Therefore, this study provides a convenient and promising nanobiocatalyst for the commercial production of γ-CD at a low cost.

## Methods

### Bacterial strains, plasmids, and reagents

*E. coli* DH5α (Takara, Japan) was used for plasmid construction and preservation. *E. coli* BL21 (DE3) (TIANGEN, China) was used to produce free γ-CGTase and γ-CGTase-PHA nanogranules. The bacterial strains, plasmids, and oligonucleotides used in this study are listed in Table 1. All primers used in this study were synthesized by GENEWIZ Company (Suzhou, China). The pCDFDuet-1 vector (Cat. No.71340–3, Novagen, USA) containing two sets of T7 promoters was used to co-express target genes. The plasmid pBHR68 harboring the *phaABC* operon from *R. eutropha* H16 [40] was kindly provided by Prof. GuoQiang Chen from Tsinghua University, China. Gene manipulation reagents, including T_4_ DNA ligase, restriction endonuclease, and Taq DNA polymerase, were all purchased from Takara (Tokyo, Japan). General cloning procedures and DNA isolation techniques were performed according to standard protocols.

**Table 1:** Bacterial strains, plasmids, and primers used in this study

Strain name	Genotype, description, or sequence	Source/References
*E. coli* DH5α	F^−^, φ80d*lacZ* ΔM15, Δ(*lacZYA-argF*)U169, *deoR, recA1, endA1, hsdR17(rK*^*−*^*, mK*^+^*), phoA, supE44, λ*^*−*^*, thi-1, gyrA96, relA1*	Takara
*E. coli* BL21(DE3)	F^−^*ompT hsdS*_B_(r_B_^−^ m_B_^−^)*gal dcm* (DE3)	TIANGEN
Plasmids		
pET22b (+)-cgt	Ampicillin resistance, pET22b ( +) derivative containing the *cgt* gene, to produce free γ-CGTase	Duan et al*.* [24]
pCDFDuet-1	Streptomycin resistance, dual T7 promoter	Novagen
pCDFD-AB	pCDFDuet-1 derivative containing the *phaAB* genes from *Ralstonia eutropha*, *phaAB* genes are under the control of the first T7 promoter	Ran et al*.* [32]
pCDFD-ABC	pCDFDuet-1 derivative containing the *phaABC* operon from *Ralstonia eutropha*, to produce PHA blank nano-granules. The *phaAB* genes are under the control of the first T7 promoter and the *phaC* gene is under the control of the second T7 promoter	Ran et al*.* [32]
pCDFD-ABC-cgt	pCDFDuet-1 derivative containing the *phaAB* genes under the control of the first T7 promoter and the *cgt* gene from *Bacillus clarkii* 7364 fused to the 5′ end of *phaC* from *Ralstonia eutropha* via a *linker* sequence under the control of the second T7 promoter, to produce PHA nano-granules decorated with γ-CGTase on the surface	This study
Primers		
phaC *Xho*I	5′-AAACTCGAGTGATGGCGACCGGCAAAGGC-3′	This study
phaC no start *Xho*I	5′-AAACTCGAGGCGACCGGCAAAGGCG-3′	This study
phaC stop *Avr*II	5′-TTTCCTAGGTCATGCCTTGGCTTTGACGTATCG-3′	This study
γ-CGTase *Bgl*II	5′-GAAGATCTAGCAAGTAATGCAACGAACG-3′	This study
γ-CGTase *Xho*I	5′-CTCGAGGGAGCCACCTCCCGATCCGCCACCAGAGCCACCTCCTCTTCGAAAACTTACAC-3′	This study

### Plasmid construction

First, the *phaAB* fragment amplified from plasmid pBHR68 was digested with *EcoR*I and *Hind*III and further inserted under the control of the first T7 promoter of the pCDFDuet-1 vector, forming plasmid pCDFD-AB. Second, the *cgt-linker* sequence containing a 5′ *cgt* gene encoding the γ-CGTase from *Bacillus clarkii* 7364 (Genbank Accession No.: AB082929) without its stop codon and including a 3′ *linker* gene encoding the peptide (GGGSGGGSGGGS) was synthesized according to the biased codons of *E. coli* by GENEWIZ Biotech Company (Suzhou, China) as a *Bgl*II/*Xho*I fragment. The resulting hybrid DNA fragment was ligated into the pCDFD-AB plasmid under the control of the second T7 promoter control, generating the pCDFD-AB-cgt plasmid. Finally, to obtain the fused γ-CGTase-phaC protein anchored on the PHA surface, the *phaC* gene was amplified using the primer pair (*phaC* no start *Xho*I)/(*phaC* stop *Avr*II) and inserted into the pCDFD-AB-cgt plasmid, generating pCDFD-ABC-cgt (Table 1). All the above-constructed plasmids were confirmed by DNA Sanger sequencing by Sangon Biotech Company (Shanghai, China).

### Production and purification of PHA granules

The pCDFD-ABC-cgt plasmid was transformed into the *E. coli* BL21(DE3) strain to produce γ-CGTase-PHA nanogranules. The production and purification procedures of the PHA nanogranules were conducted as previously described [32] with some modifications. Briefly, the glycerol stock of recombinant *E. coli* BL21 (DE3) cells was inoculated in LB medium containing 100 µg/mL streptomycin (Amresco Company, America) at 37 °C with 200 rpm orbital shaking on a rotary shaker (Zhicheng Company, Shanghai, China) for 12 h. Then the seed cultures were inoculated into modified TB-MM and LB-MM medium supplemented with 100 µg/mL streptomycin and 5 g/L glucose and cultivated at 37 °C, 200 rpm until the optical density at 600 nm (OD_600_) reached 0.6–0.8. IPTG at a final concentration of 1.0 mM was then added to induce the expression of heterogeneous proteins. Glucose was added at an additional concentration of 5 g/L after 24 h of cultivation. The basal MM medium comprised four components. Component I contained Na_2_HPO_4_·12H_2_O (9.65 g/L) and KH_2_PO_4_ (1.5 g/L), component II contained (NH_4_)_2_SO_4_ (4.0 g/L), MgSO_4_·7H_2_O (0.41 g/L), and yeast extract (1.0 g/L), component III contained Fe (III)–NH_4_–citrate (3.2 g/L) and CaCl_2_·2H_2_O (1.5 g/L) in 1 M HCl, and component IV contained ZnSO_4_·7H_2_O (100 mg/L), 25.5 MnSO_4_·H_2_O (mg/L), H_3_BO_3_ (150 mg/L), CoCl_2_·6H_2_O (200 mg/L), CuCl_2_·2H_2_O (6.8 mg/L), NiSO_4_·6H_2_O (19 mg/L), and NaMoO_4_·2H_2_O (30 mg/L) in 1 M HCl. The modified TB-MM and LB-MM medium was prepared by replacing component II of basal MM medium with the components of LB (10 g/L tryptone, 5 g/L yeast extract, and 10 g/L NaCl) and TB medium (12 g/L tryptone, 24 g/L yeast extract, 5 g/L glycerol, 2.31 g/L KH_2_PO_4_, 12.54 g/L K_2_HPO_4_), respectively.

After induction at 30 °C at 200 rpm for 48 h, cells were harvested for the purification of PHA granules. The harvested bacterial cells were washed and resuspended in Tris–HCl buffer (20 mM, pH 8.0) and disrupted on ice using a sonicator. The cell lysates (~ 15 mL) were loaded onto a glycerol gradient containing 88% and 44% (v/v) glycerol in Tris–HCl buffer. After ultracentrifugation for 2.5 h at 100,000 ×*g* and 10 °C, the PHA granules were found in the white layer between the 88% and 44% glycerol layers. The collected granules were washed three times with 20 volumes of Tris–HCl buffer and centrifuged at 100,000 ×*g* for 30 min at 10 °C. Finally, the sediment containing the PHA beads was lyophilized for 8 h, yielding purified PHA powder.

### Characterization of the PHA granules

The intracellular PHA granules were identified by transmission electron microscopy (TEM) analysis.

The recombinant cells were washed three times with PBS buffer and fixed with 2.5% glutaraldehyde in the same buffer for 12 h at 4 °C. Subsequently, the fixed cells were prepared for TEM as previously described [41].

### Analysis and quantification of attached proteins on PHA nanogranules

The concentration of total protein associated with purified PHA granules was determined by the Bradford method, and the protein profiles were routinely analyzed by SDS–PAGE using a 12% separating gel and a 4% stacking gel. The identification and quantification of the attached proteins on PHA nanogranules were performed using MS-based quantitative proteomics via isotope dimethyl labeling as previously described [25, 32].

### Production and purification of free γ-CGTase

The free γ-CGTase was produced using the recombinant *E. coli* BL21 (DE3) strain harboring the pET22b (+)-cgt plasmid as previously described [24]. The harvested bacterial cells were washed and suspended in Tris–HCl buffer (20 mM, pH 8.0) and lysed on ice using a sonicator. The free γ-CGTase was crudely separated from cell supernatant by ammonium sulfate precipitation for 12 h at 4 °C. The precipitate was collected by centrifugation at 4 °C and 12,000 ×*g*, dissolved in acetic acid buffer (pH 5.5), and dialyzed in the same buffer three times. Then, the supernatant of the protein solution was loaded onto an α-CD-Sepharose 6B affinity column equilibrated with acetic acid buffer. The free γ-CGTase was collected from the column by elution with acetic acid buffer containing 10 g/L α-CD. The eluate was then dialyzed in 10 mM PBS buffer (pH 8.0) three times and condensed to 1 mL in an ultrafiltration tube (Millipore, USA) at 4 °C and 12,000 ×*g*. The concentrated enzyme fraction was gel-filtered by a HiLoad 16/600 Superdex 200 pg column (GE Healthcare, USA) equilibrated with 10 mM PBS buffer (pH 8.0). The fractions containing γ-CGTase activity were collected and lyophilized to obtain pure γ-CGTase powder. The concentration of the purified enzyme was determined by the Bradford method using bovine serum albumin as a standard, and the purity was analyzed by SDS–PAGE.

### Enzyme assay

The hydrolysis activity of γ-CGTase was determined as dextrinizing power as previously described [24]. The cyclization activity of γ-CGTase was determined by quantifying the formed γ-CD using starch as a substrate and was detected by bromocresol green (BCG) method as previously described [5].

The turbid solution of γ-CGTase-PHA nanogranules was pretreated for homogeneous dispersion by sonication as follows: 0.1 g of dry PHA powder was added to 10 ml 50 mM Tris–HCl buffer (pH 9.0), and vortexed for 30 s, and then dispersed evenly by ultrasonic treatment (250 W, 8 s treatment followed by a 20 s pause for a total of 15 min). Afterward, the hydrolysis and cyclization activity of γ-CGTase-PHA beads were determined as previously described [5, 24].

### Effects of pH, temperature, and metal irons on γ-CGTase activity and the stability of γ-CGTase-PHA nanogranules

For the characterization of enzyme properties, the free and immobilized γ-CGTase solutions were prepared in the concentration of 10 mg/mL and 0.0568 mg/mL, respectively, to ensure that the two solutions contained equivalent concentrations of γ-CGTase protein. The optimal temperature of the immobilized γ-CGTase was determined using standard assay conditions at various temperatures (30–65 °C). Thermal-stability assays were conducted by pre-incubating the γ-CGTase-PHA nanogranules and free γ-CGTase at a series of temperatures (45–60 °C) for 6 h in 50 mM glycine–NaOH buffer (pH 9.0) before determining the residual γ-CGTase activity at 1-h intervals. The activity of the unheated enzyme was defined as 100%.

To determine the optimal pH of γ-CGTase activity, the reaction mixture was incubated at pH values ranging from 5.0 to 12.0 using different buffer systems as follows: 0.2 M citric acid–Na2HPO4 (pH 5.0–6.5), Tris–HCl (pH 7.0–8.5), glycine–NaOH (pH 9.0–11.0), and Na_2_CO_3_–NaHCO_3_ (pH 11.5–12.0) buffer. To investigate the pH stability of the γ-CGTase-PHA nanogranules and free γ-CGTase, the enzymes were pre-incubated at 50 °C for 6 h in buffers of different pHs ranging from 8.0 to 11.0 (hydrolysis) and 7.0 to 10.0 (cyclization), and the residual γ-CGTase activity was detected at 1-h intervals under standard conditions.

### The synthesis of γ-CD catalyzed by γ-CGTase-PHA nanogranules

Two percent (w/v) native cassava starch was suspended in 50 mM glycine–NaOH buffer including 1 mmol/L K^+^, Ca^2+^, and Mg^2+^ (pH 9.0) and then gelatinized with constant stirring at 70 °C. The suspension was then cooled to 50 °C and adjusted to the optimal pH by adding 2 M HCl, followed by the addition of the immobilized γ-CGTase (2000 U/g starch) for 10 h at 50 °C in a water bath shaker to synthesize γ-CD. The product mixture was then centrifuged at 8000 ×*g* for 20 min at 4 °C (Eppendorf Company, Hamburg, Germany), and the supernatant was collected for further analysis.

The produced supernatant containing liberated cyclodextrins was filtered through a 0.45 µm filter (Millipore, USA) before being analyzed by high-performance liquid chromatography (HPLC) (Agilent, USA). Three cyclodextrins purchased from Sigma company were prepared in standard solutions of 0.1 g/mL in distilled water. The analysis was conducted using a phenyl-2 HYPERSIL (4.6 mm × 150 mm) column (Thermo Fisher Scientific, USA) at 40 °C. The mobile phase was 100% aqueous solution at a flow rate of 1.0 mL/min. The detector was differential refraction k-2301 (KNAUER, Germany), and the injection volume was 10 µL. The peak area was determined by the HPLC electrical signal. The γ-CD yield (%) and the ratio of γ-CD in the product CDs were calculated with Eqs. (1) and (2), respectively, as follows:

1 $$ {\text{Yield of }}\gamma - {\text{CD}}\, = \,{\text{C}}_{{\gamma - {\text{CD}}}} *{\text{V}}/{\text{M}}*{1}00\% $$

2 $$ {\text{Ratio of }}\gamma - {\text{CD}}\, = \,{\text{C}}_{{\gamma - {\text{CD}}}} /\left( {{\text{C}}_{{\gamma - {\text{CD}}}} \, + \,{\text{C}}_{{\beta - {\text{CD}}}} } \right)*{1}00\% , $$

where C_γ-CD_ is the concentration of γ-CD (g/mL) in the product calculated by Eq. (3), V is the volume of the product solution, M is the mass of the substrate before the reaction, and C_β-CD_ is the concentration of β-CD (g/mL) in the product calculated by Eq. (4):

3 $$ {\text{C}}_{{\gamma - {\text{CD}}}} \, = \,{\text{A}}_{{\gamma - {\text{CD}}}} /{\text{A}}_{{1}} *{\text{C}}_{{1}} $$

4 $$ {\text{C}}_{{\beta - {\text{CD}}}} \, = \,{\text{A}}_{{\beta - {\text{CD}}}} /{\text{A}}_{{2}} *{\text{C}}_{{2}} , $$

where A_γ-CD_ and A_1_ are the peak areas of γ-CD in the sample and standard solution, respectively. C_1_ (g/mL) and C_2_ (g/mL) are the concentrations of γ-CD and β-CD in standard solution, respectively. A_β-CD_ and A_2_ are the peak areas of β-CD in the sample and standard solution, respectively.

### Reusability of γ-CGTase-PHA nanogranules

To assess the reusability of γ-CGTase-PHA nanogranules, a total of 20 g/L native cassava starch in 50 mM glycine–NaOH buffer (pH 9.0, including 1 mmol/L K^+^, Ca^2+^, and Mg^2+^) was gelatinized under constantly stirring at 72 °C for 10 min. Then 32.35 g/L γ-CGTase-PHA beads were added to the reaction system and incubated for 12 h at 50 °C and pH 9.0. At the end of the incubation period, the samples were centrifuged at 8000 ×*g* for 20 min, and the supernatant was collected to measure the generated γ-CD by HPLC. The sedimented γ-CGTase-PHA nanogranules were resuspended in the same reaction system as described above for the next round. The cycle was repeated eight times, and the residual enzyme activity of the nanogranules and yield of γ-CD was tested and calculated per cycle. The activity of the unused enzyme was defined as 100%.

### Supplementary Information

**Additional file 1: Table S1.** Identification and absolute quantification of fused γ-CGTase using mass spectrometer based quantitative proteomics.

## Data Availability

All data generated or analyzed during this study are included in this published article.

## Abbreviations

CD: Cyclodextrin
γ-CD: γ-Cyclodextrin
CGTase: Cyclodextrin glycosyltransferase
γ-CGTase: γ-Cyclodextrin glycosyltransferase
PHA: Polyhydroxyalkanoate
PhaA: β-Ketothiolase
PhaB: Acetoacetyl-CoA reductase
PhaC: PHA synthase
PhaP: Phasins
PhaZ: Depolymerases
*E.** coli*: *Escherichia* *coli*
TEM: Transmission electron microscopy
BCG: Bromocresol green
HPLC: High-performance liquid chromatography
SEC: Size exclusion chromatography
IPTG: Isopropyl-β-d-1-thiogalactopyranoside
MS: Mass spectrometry
